# Primary cilia as dynamic and diverse signalling hubs in development and disease

**DOI:** 10.1038/s41576-023-00587-9

**Published:** 2023-04-18

**Authors:** Pleasantine Mill, Søren T. Christensen, Lotte B. Pedersen

**Affiliations:** 1 MRC Human Genetics Unit, Institute of Genetics and Cancer, University of Edinburgh, Scotland; 2 Department of Biology, University of Copenhagen, Denmark

## Abstract

Primary cilia, antenna-like sensory organelles protruding from the surface of most vertebrate cell types, are essential for regulating signalling pathways during development and adult homeostasis. Mutations in genes affecting cilia cause an overlapping spectrum of >30 human diseases and syndromes, the ciliopathies. Given the immense structural and functional diversity of the mammalian cilia repertoire, there is a growing disconnect between patient genotype and associated phenotypes, with variable severity and expressivity characteristic of the ciliopathies as a group. Recent technological developments are rapidly advancing our understanding of the complex mechanisms that control biogenesis and function of primary cilia across a range of cell types and are starting to tackle this diversity. Here, we examine the structural and functional diversity of primary cilia, their dynamic regulation in different cellular and developmental contexts and their disruption in disease.

## Introduction

**Figure 1:**
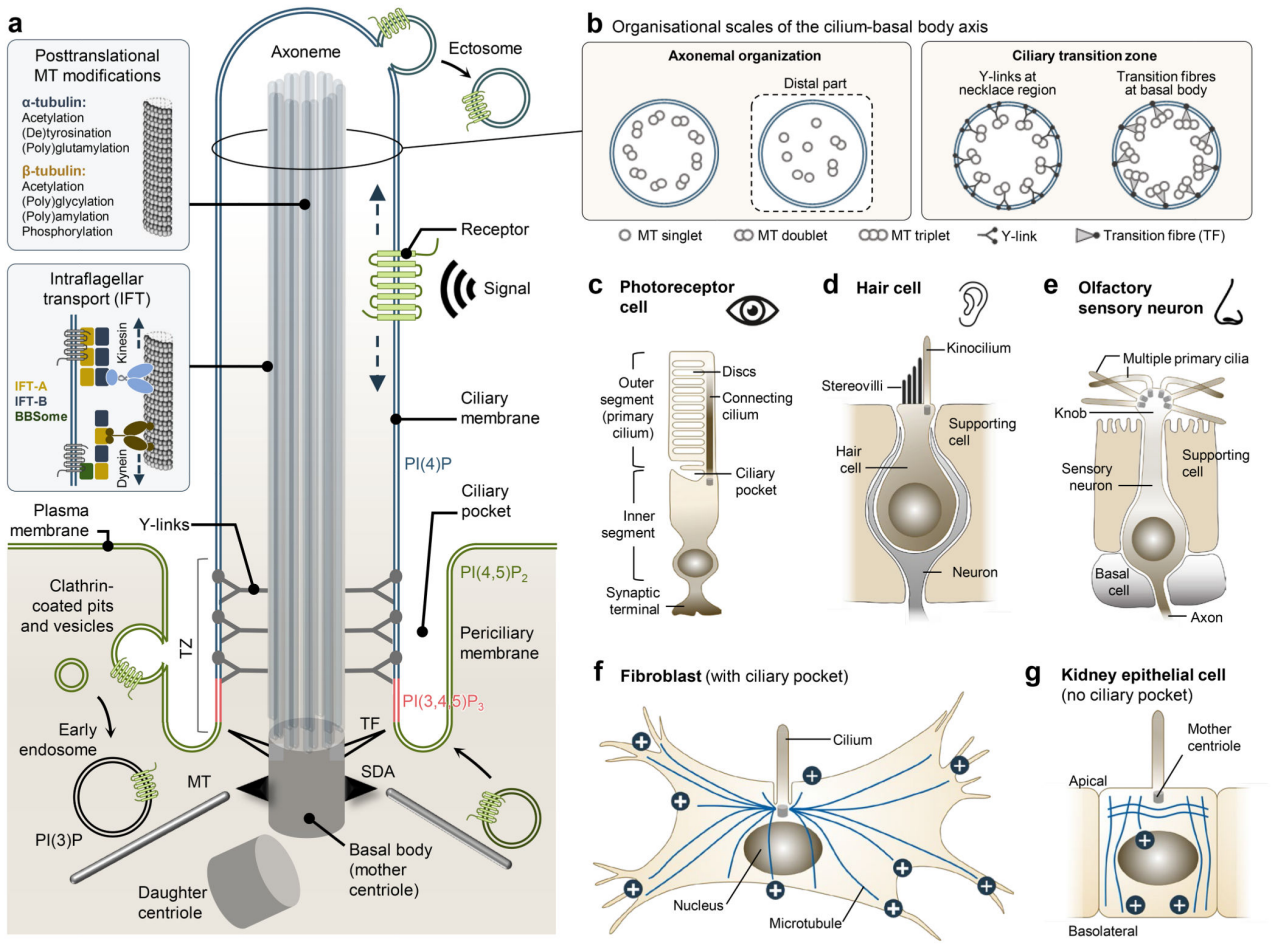
Architecture of primary cilia and main ciliary sub compartments. **a**, Schematic of a generic primary cilium showing the main ciliary subcompartments. **b**, Cross-sectional views of the cilium-basal body axis (from tip to base) showing the changes in microtubule (MT) arrangement along the axis. The panel 1b right image presented in the box for axonemal organization illustrates the compromised 9+0 MT organization of unstructured bundles of MT singlets and doublets that can be observed at the distal region of the cilium in certain cell types. **c**, **d**, **e**, Diagrams of the indicated vertebrate cell types illustrating the diverse types of non-motile, sensory cilia found in these cells. **f, g**, Depictions of a fibroblast and polarized kidney epithelial cell showing the presence and absence, respectively, of a ciliary pocket, and the different cytoplasmic MT organisation (blue lines; MT plus ends are marked with “+”) in the two cell types. Abbreviations: IFT, intraflagellar transport; SDA, subdistal appendage; TF, transition fibre; TZ, transition zone.

Primary cilia are solitary, antenna-like sensory organelles protruding from the surface of most vertebrate cell types. They consist of a microtubule core, the axoneme **[G]** , which extends from a modified centriole **[G]** called basal body **[G]** and is surrounded by a lipid bilayer membrane that is continuous with, but compositionally distinct from, the cell body plasma membrane (Figure 1a). Although they are small, representing roughly 1/200^th^ of the total surface of the cell, primary cilia are essential for development and homeostasis, as the cilium is highly enriched in receptors, ion channels and downstream effectors for several signalling pathways, including Hedgehog and G protein coupled receptor (GPCR) signalling; without this antenna-like organelle to localize to, these pathways fail to signal appropriately. Accordingly, mutations that impair ciliary biogenesis, structure or function cause deregulated signalling and lead to ciliopathies **[G]**, an overlapping spectrum of >30 human diseases and syndromes, which affect most tissues or organs in the body, including the eye, kidney, liver, brain, skeleton and heart (Table 1).

A major challenge for the field is deconvolving how genetic changes lead to the clinical phenotypes seen in patients with ciliopathies, and how defective primary cilia can give rise to isolated, single organ diseases or syndromic ciliopathies that affect multiple organs. While some types of ciliopathy-causing mutations, for example, in certain genes that cause Bardet–Biedl syndrome (BBS), can reliably predict patient phenotype^1^, in other cases a clear genotype–phenotype correlation can be challenging to establish. A prominent example is *CEP290* (OMIM 610142), mutations in which can give rise to five distinct ciliopathies, including BBS, Joubert syndrome (JBTS), Meckel syndrome (MKS), nephronophthisis (NPHP) and the related Senior-Løken syndrome (SLSN), in addition to Leber congenital amaurosis (LCA), an early-onset vision loss (Table 1). Multiple factors account for the clinical heterogeneity in patients with ciliopathies, such as the type of alleles, overlapping and cell type-dependent functions of ciliary proteins, the specific timing of ciliary dysfunction during development, and modifier genes or genetic background ^2–5^.

Currently, 247 genes are known to give rise to ciliopathies when mutated, affecting either motile cilia, primary cilia or both^2,6,7^. Most of these established ciliopathy genes encode proteins that localize to the cilium–basal body axis and are associated with so-called first-order ciliopathies, whilst genes mutated in second-order ciliopathies encode proteins that are localized elsewhere in the cell and affect cilia indirectly^2,7^ (Table 1). Classic examples of first-order ciliopathy genes are those that encode components of the intraflagellar transport **[G]** (IFT) machinery, a highly conserved microtubule-based transport system operating within cilia, which is broadly required for ciliary assembly, maintenance and function. Importantly, the detailed 3D structure of protein complexes involved in IFT has now been solved (Box 1) ^8^, which is facilitating the structural modelling of pathogenic variants to predict patient phenotypes^1^. Examples of second-order ciliopathy genes include those encoding transcription factors that regulate the expression of ciliary genes, or proteins of the secretory pathway that affect, for example, glycosylation and trafficking of integral ciliary membrane proteins ^2,7^. In addition to these established ciliopathy genes, a recent survey of the Human Phenotype Ontology (HPO) database estimated that >300 additional human disorders that are not classified as ciliopathies may have a phenotypic spectrum that includes ciliary defects. These have been referred to as ‘disorders with ciliary contribution (DCC)’; the extent of ciliary dysfunction and how it arises in these diseases remain largely unknown^7^. Thus, the ciliopathies represent an expanding number of pleiotropic human diseases for which the underlying aetiology is not fully understood.

Addressing the knowledge gap between genotype and phenotype is essential to understand the aetiology of ciliopathies, improve diagnostics and develop treatments for these diseases. Recent work has demonstrated that primary cilia exhibit remarkable structural and compositional diversity across cell types, tissues and developmental scale, which allows cilia to function as versatile signalling hubs that can fine-tune their content and signalling function according to the needs of the cell and, ultimately, the organism. Such structural and functional diversity of cilia is regulated by a series of complex and dynamic mechanisms that may, at least in part, offer a plausible explanation for the genetic and phenotypic heterogeneity observed in ciliopathies.

Here, we discuss the architectural and functional diversity of primary cilia in different vertebrate cell and tissue types. We explore how ciliary signalling function is regulated by compartmentalization and dynamic changes in the ciliary proteome in response to cellular or environmental cues and highlight how new technological developments are advancing the field. Finally, we provide examples of how human disease genetics and patient-derived stem cells can be leveraged to dissect cilia diversity *in vivo*, pointing us towards both new genes and novel functions for known cilia proteins. We do not cover motile cilia and associated ciliopathies, which have been reviewed elsewhere^6,9^.

## Architecture of primary cilia

All types of cilia consist of a membrane-covered, microtubule-based axoneme that extends directly from a basal body, which anchors the cilium to the cell body by means of transition fibres (derived from centriolar distal appendages) and subdistal appendages (Figure 1a) ^10^. At the interface between the basal body and cilium itself lies the transition zone **[G]** (TZ), a ciliary gating structure that connects the axonemal microtubules via Y-shaped assemblies to the ciliary membrane at a site known as the ciliary necklace **[G]** (Figure 1a).

### TZ composition, structure and function

A proteomic analysis of purified TZs from *Chlamydomonas* identified 115 distinct proteins, several of which are known to be mutated in human ciliopathies ^11^, including components of the three main TZ protein modules, the MKS, NPHP and CEP290 modules ^2,12–14^. Super-resolution imaging and structural biology approaches have provided insight into the molecular architecture of the TZ ^15,16^ and its spatial arrangement of individual proteins ^17–19^ and lipids ^20^. The length, structure, and composition of the TZ can vary significantly between different cell types and species ^21^. For example, the connecting cilium of mouse photoreceptors, which is equivalent to the TZ in other cilia types, is 1-1.5 μm in length, whereas the TZ of primary cilia of a human retinal pigment epithelial cell line is about 0.3 μm in length ^17,22^. There is growing evidence supporting the existence of cell type-specific differences with respect to expression and function of TZ proteins, and how these could modulate bespoke ‘gating’ functions. For example, the related proteins RPGRIP1L (OMIM: 610937; also known as NPHP8 or MKS5) and RPGRIP1 (OMIM: 605446) are both highly expressed and contribute to TZ assembly in mouse embryonic fibroblasts and kidney cell lines, but in patients and mice only RPGRIP1L mutations contribute to syndromic phenotypes, whilst RPGRIP1 mutations cause non-syndromic inherited retinal dystrophy ^23^. Similarly, when the broadly expressed *Tctn1* and *Tctn2* genes, encoding components of the MKS complex of the TZ, are knocked out in mice, striking tissue-specific differences in ciliogenesis defects and ciliary content are observed, which could explain the spectrum of features found in patients with JBTS13, who harbour mutations in *TCTN1* (OMIM: 609863) or patients with JBTS24, who harbour mutations in *TCTN2* (OMIM: 613846) ^24^. It is still unclear why these tissue-specific sensitivities exist. Furthermore, in contrast to IFT-associated protein complexes (Box 1), the detailed 3D structure of ciliary TZ protein complexes remains largely unknown.

While the phenotypic consequences of TZ gene mutations vary from mild to severe, depending on the type of allele and gene affected, most lesions in such genes result in altered ciliary protein content, thereby affecting signalling ^14^. Studies in multiple organisms and cell types have shown that the TZ, together with the transition fibres, controls the ciliary entrance and exit of both soluble and membrane-bound proteins by forming a selective diffusion barrier between the cilium and cell body. The mechanisms that allow certain proteins to cross this barrier are still not clear, but two models, which are not mutually exclusive, have been proposed: the ‘open sesame’ and ‘motorized plow’ models. The open sesame model posits that cargo binding to ciliary import factors, such as the IFT-A subcomplex, induces their privileged crossing of the TZ independently of motor activity ^14,25^. This model is supported by studies indicating that several membrane-associated proteins enter cilia independently of IFT kinesin-2 motors ^25,26^, but in most cases require interaction with the IFT-A subcomplex and associated TUBBY domain proteins TULP3 (OMIM: 604730) and TUB (OMIM: 601197) for ciliary entrance ^27–30^. According to the motorized plow model, ciliary cargoes are dragged physically across the TZ diffusion barrier by the action of IFT motors ^25^. In support of this model, anterograde IFT trains with associated axonemal/soluble cargoes assemble at the TZ prior to ciliary entrance, and mutations in a kinesin-2 motor subunit impair IFT train localization at the TZ and prevent axoneme extension ^31–34^. Conversely, several studies showed accumulation of cargoes in cilia upon disruption of specific IFT dynein 2 subunits ^35–38^. Interestingly, in ciliated sensory neurons of *Caenorhabditis elegans* lacking the dynein 2 intermediate chain subunit WDR-60, such IFT accumulations were prevented by disruption of specific TZ modules, indicating that the underpowered dynein 2 motor is unable to breach the TZ barrier unless the latter is disrupted or weakened ^38^. Moreover, inactivation of the dynein 2 heavy chain (CHE-3) or IFT-A components in worms affected TZ assembly and gating function ^39,40^, indicating that the TZ and IFT machinery reciprocally affect each other.

Importantly, whilst many of these players were key to understanding functional compartmentalization of cilia, they are also human disease genes (Table 1). The next challenge is to understand how different patient variants in seemingly core components of ciliary protein modules operating at the TZ can result in tissue-specific dysfunction, such as reported for *IFT140* or *CEP290* variants causing non-syndromic inherited retinal diseases ^41,42^. In the case of *CEP290* variants, tissue-specific isoforms seem to be at play and can be specifically targeted by therapeutics to halt or reverse visual decline^43^. Indeed, anti-sense oligonucleotide clinical trials targeting this cryptic intronic variant are underway ^44^, as was one for genome surgery which was recently halted but demonstrated proof-of-concept in patients with LCA ^45^.

### The ciliary axoneme

While all cilia are microtubule-based, the configuration of the axonemal microtubules exhibits significant structural diversity. How this is genetically programmed and how it affects cilia signalling remain open questions. A reductionistic textbook view of a primary cilium **[G]** is one of 9 microtubule doublets lacking a central pair, termed a ‘9+0’ configuration versus the ‘9+2’ configuration of motile cilia axonemes (Figure 1a, b). However, as the detailed ultrastructure of primary cilia from more and more cell types is being uncovered, aided by advances in imaging technologies such as serial section electron tomography (SSET) ^46^, cryo-electron tomography (cryoET) ^47^ and focused ion beam scanning electron microscopy (FIB-SEM) ^48,49^, it becomes clear that substantial deviations from this canonical ciliary architecture exist. At one extreme are the vertebrate rod and cone photoreceptors whose outer segment is a specialized primary cilium composed of numerous opsin-containing membranous discs (rods) or lamellae (cones), which is bridged to the inner segment by a modified TZ called connecting cilium (Figure 1c). The distal end of the connecting cilium extends into an axoneme that terminates with an elongated microtubule singlet segment missing the B subfibre found in canonical axonemal outer doublet microtubules ^50^. Atypically shaped non-motile cilia with elaborate membranous appendages and/or distal axonemal singlet extensions are also found on sensory neuronal cells of nematodes ^51^, whereas the primary ciliary axoneme of mammalian kidney epithelial cell lines displays ‘9 + 0’ microtubule configuration only at the base, with the middle and distal regions being comprised largely by an unstructured bundle of microtubule singlets and actin filaments (Figure 1b) ^46,47,52^. Similar microtubule thinning towards the axoneme tip has been reported in zebrafish embryos using ultra-expansion microscopy (UExM) ^53^. Additional examples of vertebrate cilia with diverse axoneme structure include the ‘9+2’ structure of kinocilia of inner ear hair cells that retain outer dynein arms; these modified ‘primary’ cilia play key roles in hair cell morphogenesis and maintenance, hence act ‘passively’ to allow mechano-electrical transduction to sound (Figure 1d) ^54,55^. In mammals, olfactory sensory neuron cilia are also ‘9+2’ (Figure 1e), but are immotile as they lack dynein arms ^56,57^. The functional significance of maintaining a central pair remains unclear. There are also examples of ‘9+0’ sensory cilia with evidence of cilia motility, including multiciliated choroid plexus epithelial cells involved in sensing cerebral spinal fluid ^58^, and recently in glucose-dependent motility of β-cell cilia in pancreatic islets, which are necessary for insulin secretion ^59^. As long as there are rules, there will clearly be exceptions to this ‘two’ cilia state.

The molecular basis for the structural diversity of cilia is not fully understood, but studies in a range of model organisms are starting to provide some clues ^60^. First, employment of ‘accessory’ kinesins that work in concert with the canonical anterograde IFT motor, heterotrimeric kinesin-2, may promote assembly of extended distal axonemal singlet microtubules in specific subsets of cilia. This is exemplified by the *C*. *elegans* homodimeric kinesin-2 motor, OSM-3, which mediates assembly of distal axonemal singlet segments in amphid channel cilia but not amphid wing cilia ^61^. Similarly, the vertebrate OSM-3 homologue KIF17 seems to specifically modulate the assembly of olfactory cilia and photoreceptor outer segments, but not other cilia types ^62,63^. Second, confinement of IFT trafficking to specific axonemal doublet (or singlet) microtubules, as observed in *Chlamydomonas reinhardtii* ^64^ and *Trypanosoma brucei* ^65^, could allow for structural or molecular specialization of the microtubules not utilized as tracks for IFT ^60^. Such structural and functional diversification of microtubules within the same axoneme may be influenced by the ‘tubulin code’: either specific tubulin isotypes ^66,67^ or post-translational modifications that regulate microtubule dynamics (Figure 1a) ^68,69^. These reversible marks play critical roles in the timing of cilia assembly and disassembly as well as function of multiple cilia types, including vertebrate sperm flagella ^70^, photoreceptor outer segments ^71^, and subsets of *C*. *elegans* neuronal sensory cilia ^72,73^. Finally, cell type-specific expression of IFT cargoes ^74^ or of centriolar and TZ components ^18,23^, which provide the foundation for the axoneme and regulate ciliary protein content, could also influence ciliary structural diversity. Importantly, since the IFT system not only mediates intraciliary transport of axonemal building blocks, but also regulates ciliary transport of membrane-bound and soluble signalling molecules, as discussed below, the above-described mechanisms are likely to impinge on ciliary membrane composition and signalling function.

### The ciliary membrane

**Figure 2:**
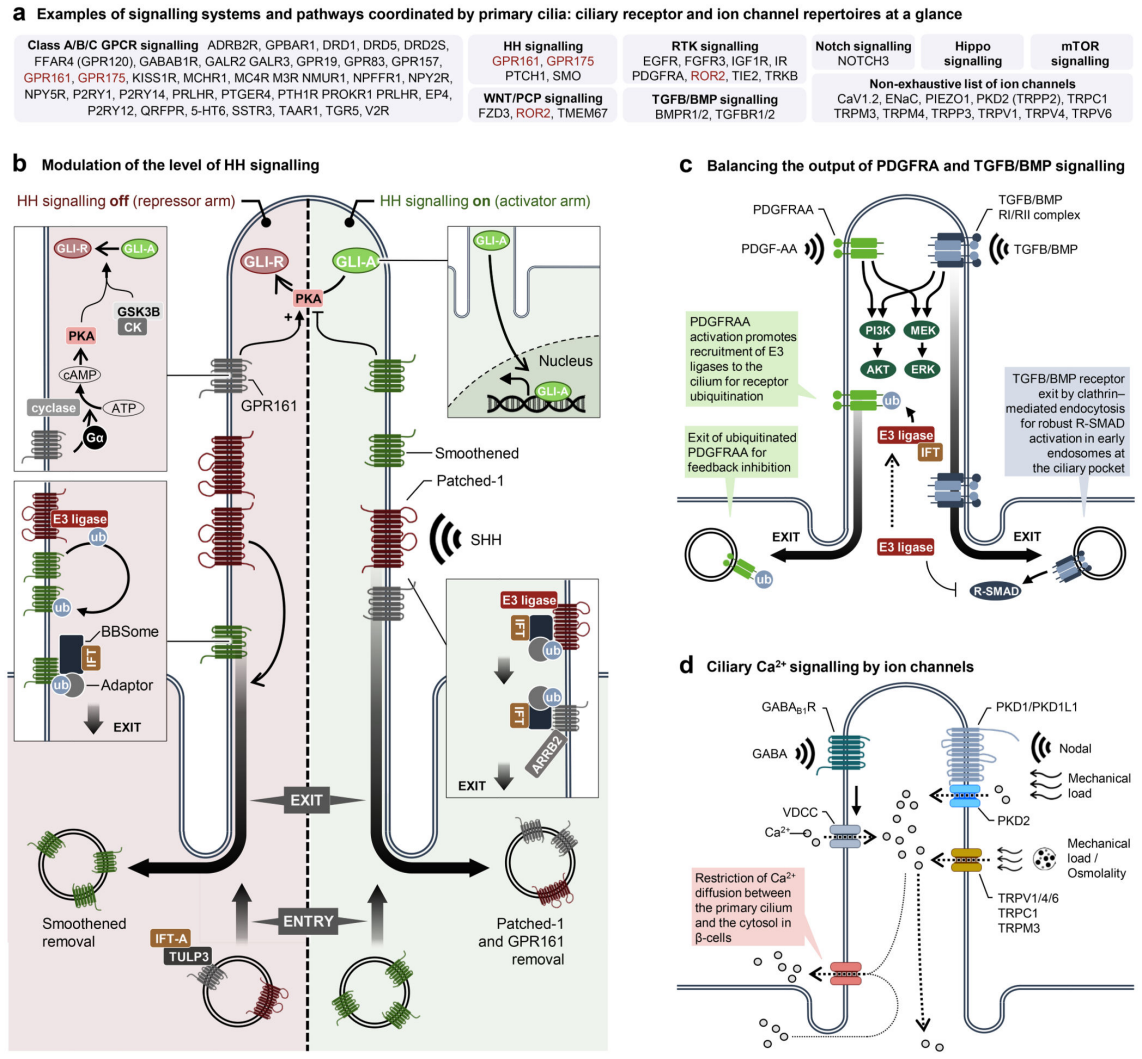
Overview of signalling pathways coordinated by primary cilia. **a**, Overview of receptors and ion channels in ciliary signalling pathways. Receptors in red are listed twice, as they can be categorized in more than one signalling system. **b**, Overview of Hedgehog (HH) signalling. In the absence of sonic hedgehog (SHH) (in the repressor arm of HH signalling), the receptor patched-1 (PTCH1) is enriched in the ciliary membrane, preventing ciliary enrichment of smoothened (SMO) through WWP1 (E3-ligase)-mediated ubiquitination and ciliary exit by retrograde IFT. The class A GPCR, GPR161, is targeted to the cilium by tubby-like protein 3 (TULP3) and IFT-A to activate adenylate cyclases via G-proteins (Gα), leading to increased ciliary levels of cAMP. cAMP activates protein kinase A (PKA), which in complex with glycogen synthase kinase 3 β (GSK3β) and casein kinases (CK) promotes the limited proteolytic cleavage of full-length and activator versions of GLI2/3 transcription factors (GLI-A) into their repressor form (GLI-R). In the presence of SHH (in the activator arm of HH signalling), PTCH1 and GPR161 exit the cilium, allowing enrichment of ciliary SMO, which promotes formation of GLI-A. Exit of PTCH1 and GPR161 similarly relies on their ubiquitination and removal by BBSome-assisted retrograde IFT; GPR161 ubiquitination being controlled at the level of beta-arrestin 2 (ARRB2). Both GLI-A and GLI-R translocate from the cilium into the nucleus to induce and repress transcriptional activation of HH target genes, respectively. **c**, Overview of ciliary control of platelet-derived growth factor α (PDGFRα) and transforming growth factor β (TGF-β)/bone morphogenetic protein (BMP) signalling. Following activation of PDGFRα and downstream signalling via PI3K-AKT and MEK1/2-ERK1/2 pathways, E3 ligases of the CBL family ubiquitinate the receptor for internalization and feedback inhibition. TGFB/BMP signalling operates in the cilium via both canonical (R-SMAD) and non-canonical (e.g. PI3K-AKT and MEK1/2-ERK1/2 pathways). Robust canonical signalling relies on ciliary exit of activated TGFB receptors to activate R-SMADs, which are inhibited by E3-ligase SMURF1 at the ciliary pocket. **d**) Examples of stimulation modes (chemosensation, mechanosensation and osmolality) for ciliary Ca^2+^ signalling regulated by GPCRs and ion channels. Please see main text for further details. Abbreviations Ub: ubiquitination.

The ciliary membrane is connected to the plasma membrane via the periciliary membrane **[G]**, which in some cell types is invaginated, forming a ciliary pocket **[G]** that surrounds the proximal region of the cilium (Figure 1a). Other cilia types including vertebrate photoreceptor outer segments and a subset of *C*. *elegans* neuronal cilia feature elaborate membrane extensions at their distal end^50,51^. Despite being continuous with the cell body plasma membrane, the ciliary membrane has a different composition of proteins and lipids that function in signalling (Figure 2a), and which localize dynamically to cilia in response to cellular and environmental cues^25,75^. Indeed, morphology^76^ or length^77^ of cilia themselves can be altered by these signalling cues, underscoring the dynamic and highly responsive nature of these organelles.

#### The ciliary membrane proteome

The best studied examples of ciliary membrane proteins include the autosomal dominant polycystic kidney disease (ADPKD) gene products polycystin-1 (PKD1; OMIM: 601313) and polycystin-2 (PKD2; OMIM: 173910), which form a cilium-localized heterodimeric receptor-cation channel complex essential for preventing cystogenesis ^78–81^, and the Sonic Hedgehog (SHH) co-receptor Patched-1 (PTCH1) (OMIM: 601309) and the class F GPCR, Smoothened (SMO) (OMIM: 601500), which accumulate in cilia in the absence and presence of SHH, respectively ^82–84^ (Figure 2b). Without cilia to localize to, while still being expressed normally, these signalling components no longer function properly, leading to characteristic fibrocystic features (in the case of ADPKD) or patterning defects (in the case of SHH signalling defects) observed in patients with ciliopathies. Intriguingly, even within the cilium itself, domains of protein organization may exist. Recent reports of microdomains of PKD2 localization enriched along the dorsal surface of immotile nodal cilia could explain how mechanosensory responses to directional flow exist ^85,86^.

Whilst less is known about how they signal downstream, numerous other receptors and ion channels have been reported to localize to the primary cilium in a dynamic and context-dependent manner, including a growing number of G protein coupled receptors (GPCRs) ^87^, receptor tyrosine kinases (RTKs) and TGFB/BMP receptors ^88^ (Figure 2a). Recent advances in cilia-targeted proximity-labelling and proteomics approaches have expanded the repertoire of cilia-localized receptors, ion channels and downstream effectors, and revealed how this may change in response to external signalling cues and/or mutations in specific ciliopathy disease genes, for example, BBS genes ^89–94^. However, to date the ciliary membrane/signalling proteome has been studied for only a handful of vertebrate cell types, including the IMCD3 kidney epithelial cell line ^89,91^, mouse fibroblast NIH3T3 cells ^93^ and mouse and zebrafish photoreceptor outer segments ^90,94^. In the future, it will be important to compare cilia proteomes across different cell types, both *in vitro* and *in vivo*, and during different cell differentiation and developmental stages.

The mechanisms controlling dynamic spatiotemporal localization of specific receptors and other signalling proteins to the cilium are complex and involve gating by the TZ (see: TZ composition, structure and function), a specialized ciliary import system for lipidated cargoes, and components of the IFT system (Box 1), which interacts with various adaptors to ferry specific integral or peripheral membrane proteins into or out of the organelle. During ciliary membrane protein import the IFT-A subcomplex binds to the TUBBY domain proteins TULP3 and TUB, which regulate the ciliary localization of a broad range of integral membrane proteins, including GPCRs and polycystins ^27–30^. TULP3 and TUB bind to phosphoinositides via their C-terminal TUBBY domain ^27,28^ and the IFT-A complex binds directly to phosphatidic acid and ceramide ^95–97^. Moreover, purified IFT-A was shown to bind to the ciliary targeting signal in SSTR3 ^98^. Direct binding of TULP3/TUB to transmembrane cargoes remains undocumented, but a recent study indicated that TULP3 binds directly to ARL13B (OMIM: 608922) ^29^, an atypical GTPase that is highly enriched in cilia and mutated in JBTS ^99,100^. ARL13B is palmitoylated ^101^ and TULP3 is required for ciliary import of ARL13B as well as several other lipidated cargoes, including farnesylated INPP5E (OMIM: 613037) and myristoylated NPHP3 (OMIM: 608002) ^29,102–104^. In this context, ARL13B functions as a GEF for the small GTPase ARL3 (OMIM: 604695), and activated, ARL3-GTP promotes release of lipidated cargoes from their carrier proteins PDE6δ (OMIM: 602676) or UNC119/UNC119B (OMIM: 604011) causing their release into cilia ^105^.

In analogy with ARL3-dependent ciliary import of lipidated cargoes, the small GTPase RABL2B (OMIM: 605413) was shown to promote ciliary entrance of IFT trains in a GTP-dependent manner^106^. Shortly after IFT trains enter the ciliary compartment, GTP-bound RABL2 is inactivated by IFT81-IFT74, which function as a GAP to enhance GTP hydrolysis and inactivate RABL2 ^107^. While a GDP-locked RABL2 variant failed to promote ciliogenesis, a GTP-locked variant caused aberrant accumulation of the BBSome **[G]** and associated cargoes in cilia ^106,108^, presumably owing to impaired dissociation of constitutively active RABL2 from the IFT machinery that prevents binding of the latter to BBSomes at the ciliary tip ^107^. Indeed, the BBSome is a well-described membrane cargo adaptor for the retrograde IFT machinery that binds to phospholipase D and various ubiquitinated transmembrane cargoes, including the GPCRs SSTR3 and SMO, to promote their exit from cilia ^25,34,36,109–111^. In this context, the BBSome seems to employ its own adaptor, the ancestral endosomal sorting complexes required for transport (ESCRT) protein TOM1L2 (OMIM: 615519), to facilitate interaction with ubiquitinated cargoes ^111^. Not surprisingly, mutations in BBS genes cause profound changes in the ciliary membrane proteome, as revealed using cilia-targeted proximity-labelling and proteomics approaches ^36,89,90,94^.

#### Ciliary lipid composition

Phosphoinositides (PIPs) are lipid signalling molecules that coordinate multiple membrane-associated molecular events. In primary cilia, high spatial organization of PIPs is observed; the ciliary membrane is enriched for PI(4)P, whereas the TZ and plasma membrane contain primarily PI(3,4,5)P_3_ and PI(4,5)P_2_, respectively (Figure 1a) ^20,25^. This boundary of distribution is highly regulated by specific cilia-localized lipid homeostatic enzymes, such as the ciliopathy gene product and PI(3,4,5)P_3_/PI(4,5)P_2_-specific phosphatase INPP5E ^20,112-116^, and is critical for regulating ciliary localization of various signalling receptors, including multiple GPCRs, which rely on the PI(4,5)P_2_- and IFT-A associated membrane adaptors TUB and TULP3, for recruitment to cilia ^28^. Other lipid species with critical functions in cilia include ceramides, which are essential for ciliogenesis in organisms ranging from *Chlamydomonas* to humans ^96,117^, and various sterols, which in vertebrates play essential roles in regulating GPCR-based signalling including Hedgehog^118^. Notably, while the lipid composition of the ciliary membrane affects its interaction with the IFT machinery thereby influencing ciliary protein import or export ^28,96^, perturbations in IFT or its associated membrane cargo adaptors, such as the BBSome, may conversely affect ciliary lipid composition. For example, a proteomics and lipidomics analysis of isolated photoreceptor outer segments from wild type and *bbs1* mutant zebrafish showed enrichment of cholesterol and proteins involved in lipid homeostasis in outer segments of the mutants, indicating a critical role for the BBSome in regulating outer segment lipid homeostasis ^94^. Similarly, loss of BBS4 in *Chlamydomonas* caused altered composition of several lipid species within cilia, owing to abnormal accumulation of phospholipase D in the mutant cilia ^36^. Despite recent advances, the lipid composition of most vertebrate primary cilia types remains obscure owing to technical challenges associated with purifying primary cilia from such cells, combined with a paucity of reliable molecular probes for fluorescence imaging of most lipids ^118^.

#### Ciliary membrane biogenesis and homeostasis

Biogenesis of the ciliary membrane begins during initiation of ciliogenesis, which for vertebrate primary cilia can occur via two distinct pathways depending on the cell type. Mesenchymal cell types such as fibroblasts employ an intracellular pathway, in which ciliary membrane biogenesis is initiated by attachment and fusion of Golgi-derived vesicles to the mother centriole distal end before centriole docking at the plasma membrane ^119^. A similar intracellular pathway was reported for the biogenesis of vertebrate photoreceptor outer segments ^120^, whereas polarized kidney epithelial cells use an extracellular ciliogenesis pathway, whereby the mother centriole docks at the plasma membrane prior to ciliary membrane and axoneme extension ^119^. In the latter case, the midbody remnant and associated specialized membranes are believed to play a key role in initiating ciliary membrane outgrowth ^121^. For both pathways, proteins and lipids required for further ciliary membrane growth are transported in vesicles from the endoplasmic reticulum/Golgi towards the ciliary base, where vesicles are exocytosed and incorporated into the growing ciliary membrane. Many active players, for example, the small GTPases RAB8 and RAB11 and their associated effectors and regulators, are involved in such transport ^119^, some of which act in a cell type-dependent fashion. For example, the small GTPase RAB34 specifically promotes ciliary membrane biogenesis in the intracellular pathway but not the extracellular pathway ^122,123^. Considering the fundamentally different organization of cytoplasmic microtubules, along which vesicular transport of ciliary components occurs, in cell types that form cilia intracellularly versus extracellularly (Figure 1f, g), such cell type-specific requirement for ciliogenic factors is not surprising.

In addition to the enrichment processes described above, cilia also require a means to fine-tune concentration of these components to a ‘Goldilocks’ point for optimal signalling. It has been proposed that these enrichment mechanisms are counterbalanced by endocytosis at the ciliary pocket ^124,125^ and/or budding of ectosomes **[G]** or extracellular vesicles from the ciliary or periciliary membrane (Figure 1a) ^126^. While it is not yet clear whether cilia-associated endocytosis and ectosome shedding operate separately or in parallel, both processes would allow cilia appropriate content regulation by cell type and environmental conditions.

## Cilia-dependent signalling

The dynamics of ciliary protein composition translate into a remarkable flexibility in the sensory capacity of primary cilia (Figure 2a), enabling the translation of quite diverse signalling inputs via elaborate signalling networks to process information in time and space ^127^. A prominent example includes that of the GPCR family, which probably functions in all types of primary cilia, albeit in a highly cell type-specific manner, to orchestrate diverse processes during development and in tissue homeostasis. GPCRs from different classes control light detection in photoreceptor cells; odorant sensation in olfactory sensory neurons; cognitive processes in the brain; and energy homeostasis and appetite via the concerted communication between multiple organs and tissue cell types, including, but not limited to, neurons in the arcuate nucleus of the hypothalamus, pancreatic islet cells, cholangiocytes and adipose tissue precursor cells ^128–130^.

### Ciliary coordination of Hedgehog signalling

To ensure signalling flexibility, cells have evolved diverse mechanisms to monitor and fine-tune the temporal localization and interaction of regulatory proteins within the cilium-basal body axis that orchestrate cell type-specific signalling outputs in different developmental or environmental contexts ^127^. Perhaps the best described pathway system to be controlled by such mechanisms is Hedgehog signalling (Figure 2b), in which the concerted regulation of receptor trafficking and activity is orchestrated in part by unique lipid compositions of spatially distinct ciliary membrane domains ^20,25,118^.

In the absence of SHH, the cilium is enriched in PTCH1 and the class A GPCR GPR161 (OMIM 612250), which in combination form a Hedgehog signalling repression machinery that promotes the proteolytic cleavage of GLI2/3 transcription factors into their repressor forms (GLI-R), thereby preventing expression of Hedgehog target genes such as *GLI1*. In this scenario, GPR161 activates cAMP-dependent kinase (PKA), which in concert with GSK3B and CK phosphorylates GLI2/3 for their processing, while PTCH1 prevents ciliary accumulation of SMO, which counteracts proteolytic cleavage of GLI2/3. Binding of SHH to PTCH1 leads to ciliary removal of both PTCH1 and GPR161, allowing SMO to enter the ciliary compartment and inhibit PKA activity via its C-terminal PKA inhibitor (PKI) motif that functions as a decoy substrate sequence to physically block the active site of PKA and thereby switches off its enzymatic activity ^131^. This allows GLI2/3 to stay in their activator forms (GLI-A), which can traffic to the nucleus for target gene expression (Figure 2b). Furthermore, PKA inhibition in the activator arm of Hedgehog signalling was suggested to be controlled at the level of ciliary GPR175 (OMIM 608336) entry, which decreases cAMP production ^132^.

### Regulation by ubiquitination

While molecular and mechanistic insights into the dynamics of ciliary signalling in different cellular, developmental or environmental contexts, and the implications thereof in disease, are not yet fully understood, emerging evidence points to a critical function of E3 ubiquitin ligases in coordinating ciliary signalling outputs ^133^. In the repressor arm of Hedgehog signalling, this is in part controlled at the level of SMO ubiquitination ^110,134^ by the WW domain E3 ligase, WWP1, which is brought into the cilium by PTCH1. This regulatory step allows SMO to interact with the BBSome for removal by retrograde IFT ^135^ (Figure 2b). Similarly, exit of PTCH1 and GPR161 in the activator arm of Hedgehog signalling relies on their ubiquitination. GPR161 ubiquitination is controlled by beta-arrestin 2 (ARRB2) ^134^, which is recruited to the cilium upon Hedgehog stimulation to remove GPR161 ^98,136^ in concert with TOM1L2, an adaptor for BBSome-mediated retrieval of Ub^K63^-tagged GPCRs from cilia ^111^. In these conditions, assembly and function of the BBSome complex rely on its own ubiquitination carried out by GPCR-cAMP-mediated mono-ubiquitination of BBSome subunits by the RING E3 ubiquitin ligase, PJA2 ^137^. PTCH1 exit is co-regulated by the combined action of E3 ligases of the HECT domain family, SMURF1/2 ^138^, and the endocytic adaptor protein NUMB, which when mutated results in attenuation of Hedgehog signalling and developmental defects, such as reduced size of the cerebellum ^93^. Furthermore, activation of Hedgehog signalling relies on ciliary localization of the CTLH E3 ubiquitin ligase complex, which when mutated in *Xenopus laevis* causes ciliopathy-like phenotypes, including, but not limited to, neural patterning defects ^139^. Similarly, ciliary modulation of downstream pathways in RTK and TGFB/BMP signalling (Figure 2c) ^140–142^ is coupled to the dynamic translocation and function of E3 ligases in the cilium-basal body axis. The timely feedback inhibition of PDGF-AA signalling proceeds through ciliary recruitment of CBL E3 ligases of the RING finger family, followed by ubiquitin-mediated internalization of its receptor, PDGFR-A (OMIM 173490) ^143^, while SMURF1 E3 ligase at the ciliary pocket region inhibits activation of R-SMAD transcription factors in TGF/BMP signalling during heart development ^144^ (Figure 2c).In addition, cilia assembly, length control and resorption are controlled by dynamic networks of E3 ubiquitin ligases and deubiquitinases ^133,145,146^, and proteomic approaches ^147^ have delineated ciliary ubiquitinomes, which mark cell type-specific signatures of ubiquitin-mediated processes, thereby contributing to the understanding of diversity in function of primary cilia across different cell types and tissues.

### Ciliary Ca^2+^ signalling

Ciliary ion channels contribute to both osmo-, mechano- and chemosensory capabilities in various developmental and homeostatic contexts. In some cases, ion channel activity is controlled at the level of ciliary GCPRs^130^, such as in insulin-secreting β-cells of the pancreatic islet of Langerhans, where γ-aminobutyric acid-mediated activation of the class C GPCR GABA_B1_ receptor (GABAB1R) (OMIM 603540) promotes Ca^2+^ influx via L-type Ca^2+^ channel (VDCC) within the cilium proper^148^ (Figure 2d). In other cases, primary cilia sense mechanical loads, signalling molecules and/or changes in extracellular osmolality through various Ca^2+^ channels of the TRP family^149–151^ (Figure 1d). Through diverse mechanisms, primary cilia thus contribute to the orchestration of developmental processes, such as in left–right (LR) symmetry breaking during early embryogenesis ^85,86,152^, and in safeguarding function and remodelling of urinary, cardiovascular and musculoskeletal systems ^153^. While leftward fluid flow provided by rotating cilia in the pit of the LR organizer (LRO) during early embryogenesis is both necessary and sufficient to define the left side of the embryo in most vertebrates, including the mouse ^154,155^, the mechanisms by which this flow is interpreted into later asymmetrical placement and patterning of the internal organs and associated vasculature have been the subject of much debate over the past 25 years ^156^. Recent work using optical tweezers and advanced imaging techniques in mouse and zebrafish supports the conclusion that primary cilia at the left side of the LRO function as mechanosensors that convert the biomechanical forces of the flow into Ca^2+^ signals via PKD2 ^85,86^, whereas the accumulation of the PKD1L1 polycystin channel at the left mouse LRO margin may provide a chemosensory channel for Nodal-mediated Ca^2+^ signalling in LR determination ^152^. It is therefore plausible that multiple mechanisms may act in concert via primary cilia to translate nodal flow, which when defective causes heterotaxy, including, but not limited to, cardiac laterality defects ^157,158^. Indeed, emerging evidence suggests that primary cilia combine chemosensory inputs with mechanical loads, such as in the detection of low-flow forces by primary cilia during development of the retinal vasculature, which sensitizes endothelial cells to BMP signalling ^159^.

## Cilia dynamics in development and disease

**Figure 3:**
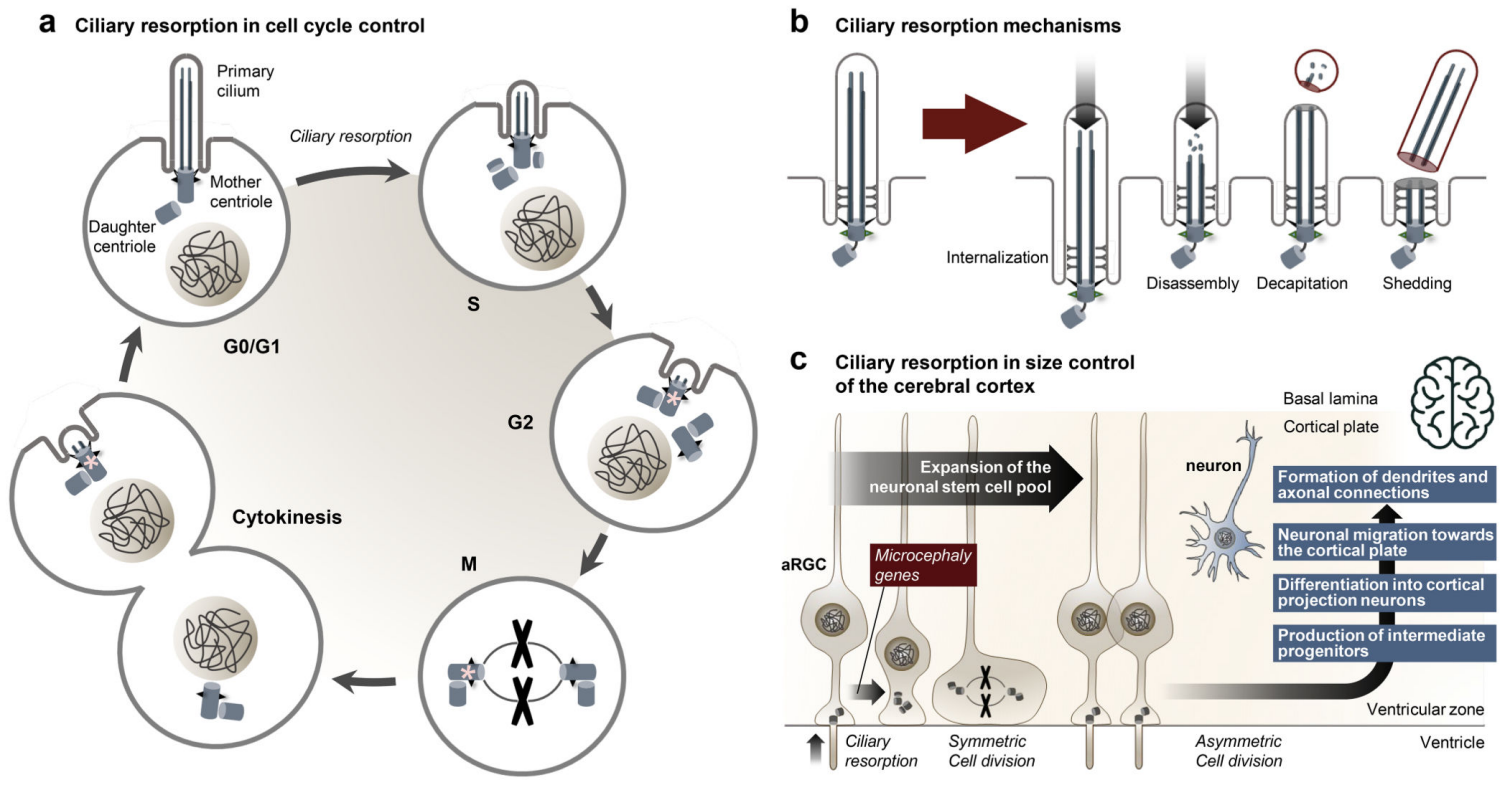
Ciliary dynamics. **a**, Assembly and disassembly of primary cilia are tightly coordinated with the cell cycle. A single primary cilium assembles from the mother centriole in G0/G1 phase, and the cilium is resorbed during the G1/S and M phases to liberate duplicated centrioles for mitotic spindle pole formation. At the end of cytokinesis, the daughter cell inheriting the oldest mother centriole (marked with an asterisk), will begin forming a new primary cilium prior to the other daughter cells. **b**, Schematic illustration of different modes by which a cilium can be resorbed. **c**, During development of the cerebral cortex, apical radial glia cells (aRGC) primary cilia, which project into the ventricular lumen, are resorbed to allow expansion of the neural stem cell pool by symmetric cell divisions. By asymmetric cell divisions, aRGCs subsequently form intermediate progenitors that differentiate into cortical projecting neurons that migrate towards the cortical plate to form the dendrites and axonal connections. Consequently, dysfunction in the timely resorption of aRGC primary cilia is linked to proliferation–differentiation decision defects and reduced size of the cerebral cortex such as in microcephaly.

Unlike most organelles, cilia and the centrioles from which they are templated, are tightly regulated by the cell cycle. Cilia transit through a sequence of assembly, elongation and resorption coordinated by the centrosome **[G]** ^160^, with the latter being required to free up the centrioles for mitotic spindle pole formation (Figure 3). The molecular principles of orchestrating these transitions have been well defined in cell culture systems, generally using synchronization through serum starvation or addition ^161^. This reductionist approach has enabled the identification of cilia-linked growth factor pathways activated by serum, including RTKs ^88^ and lysophosphatidic acid receptors (LPAR) ^162^, the latter shown recently to trigger cilia disassembly and promote neurogenesis *in vivo* ^163^. In some cases, these mechanisms have not translated well *in vivo*. For example, the *in vivo* role of the second phase of the bi-phasic cilia resorption described in cell lines to depend on HDAC6-mediated axonemal tubulin de-acetylation ^164^ is unclear. Knock-out *Hdac6* mice have no phenotype, but a mutation affecting *HDAC6* post-transcriptional stability in humans (OMIM: 300272) could have a potentially ciliopathic phenotype ^165^. Nevertheless, defects in timely cilia resorption have been reported, for example, in patient fibroblasts, neuronal progenitors or brain organoids from patient induced pluripotent stem cells (iPSCs) with mutations in *CPAP* (OMIM: 609279), *WDR62* (OMIM: 613583) or *RRP7A* (OMIM: 619449), resulting in depletion of the neuronal stem cell pool in the cerebral cortex leading to primary microcephaly (MCPH) ^166–168^ (Figure 3c). As such, not only the availability of a cilium to signal but also the ability to dismantle it, through gradual resorption, rapid excision or a combination of both ^169^, seems key to progenitor fate decisions and subsequent steps in tissue morphogenesis.

### Cilia dynamics in proliferating tissue

Intricately linked with the cell cycle and cell differentiation state, primary cilia may not always be present on a cell in a growing tissue at a given moment. In a proliferative tissue, these dynamics would affect a cell’s signalling competence. Indeed, the daughter cell that inherits the ‘older’ mother centriole may become signalling competent first by assembling a cilium ahead of its sib ^170–172^. This cellular heterogeneity adds an extra layer of complexity as to how a transient organelle, which is required for mitogenic signalling including Hedgehog signalling, responds if it is only present for a portion of the cell cycle. Indeed, cilia have been shown to persist into S phase in a Hedgehog-responsive medulloblastoma cell line ^173^, in chick neural tube progenitors into late G2 *in vivo* ^174^ and on S/G2 cells in all embryonic cell types examined in *Arl13b-Fucci2* mice ^172^. How Hedgehog can instruct robust developmental decisions may involve a ‘mitotic memory’ — a means to ‘remember’ signalling between ciliated phases in proliferating tissues —to deal with the variability of having a cilium in primary cerebellar granular precursors whose proliferation drives foliation of the developing cerebellum ^173^. As many developmental decisions are made in response to Hedgehog, determined by levels and length of time of exposure to ligand, this work connects the need for robust signalling with a dynamic organelle necessary to drive the growth of the developing brain, face and limb bud, systems often affected in ciliopathies ^175^.

More than just presence or absence, we do not understand how cilia content may change with the cell cycle, although evidence suggests this occurs for phosphoinositide content ^176^ and signalling competence in subsequent interphases ^173^. Moreover, specific and rapid remodelling of the cilia proteome occurs in the timescale of tens of minutes in response to Hedgehog ligand ^92^, but how the sensitivity and responsiveness may change with cell cycle stages remains unclear. These observations raise questions, particularly in rapidly dividing tissues or cancers, as to the physiological consequence of having a cilium at different stages of the cell cycle. Future studies, possibly combining organelle-specific proximity labelling techniques with cell cycle biosensors, will be needed to determine whether cilia can function as effective ‘signalling organelles’ if present regardless of cell cycle stage, or whether their contents and signalling competence are regulated in a cell cycle-specific manner.

### Cilia dynamics during differentiation

Over longer periods, in this case developmental time, ciliation status or axoneme configuration may change within a tissue. Both olfactory ^56^ and choroid plexus ^58^ cilia transit from motile to sensory functions soon after their birth. Developing from a population of ciliated bipotential hepatoblast progenitor ^177^, the adult liver is composed mostly of hepatocytes, which are predominantly non-ciliated, and cholangiocytes of the biliary tract that are highly ciliated ^178,179^. Interestingly, mitogens, such as SHH, play key early roles in the expansion of the progenitors, inhibiting their hepatocyte lineage commitment ^180,181^. This finding suggests that temporal restriction of Hedgehog activation via cilia status is key for cell fate. In contrast to hepatocytes, cholangiocytes have very long cilia, which are proposed to function as mechanosensors (detecting PKD1/2-mediated flow) ^182^, osmosensors (sensing TRPV4-regulated hypotonicity) ^183^ and chemosensors (identifying purigenic receptor P2Y12 (OMIM: 600515) and bile acid receptor TGR5 (OMIM: 610147) signalling) ^184^ to regulate cholangiocyte homeostasis and the biophysical properties of bile. Consistent with roles for cilia across liver development and health, mutations in cilia genes are reported in congenital anomalies such as biliary atresia ^185^ and fibrocystic liver diseases ^186^, whilst cilia defects are reported during chronic inflammation and cholangiocarcinoma ^187^. In addition, primary cilia of pre-adipocytes are configured to orchestrate multiple signalling pathways that in different environmental contexts balance differentiation decisions of cells ^129^, including, but not limited to, IGF-1R (OMIM: 147370) ^188^ and class A GPCR signalling, the latter expanding white adipose tissue via omega-3 fatty acid-mediated activation of FFAR4 (GPR120, OMIM: 609044) ^189^. Understanding cilia states and their changing configurations and functions during differentiation remains a key challenge to interpreting disease phenotypes.

### Rewiring of signalling caused by ciliary loss

Cilia loss as a result of mutation can result not only in dampening of signalling cascades that run through cilia but also misactivation of signalling responses normally held in check by an operational cilia axis. In cholangiocytes, ciliary loss drives activation of alternative signalling pathways either due to relocalization of or inhibited interaction with signalling molecules. This includes TGR5, a typically ciliary GPCR that when localized to the apical membrane alters the interaction with inhibitory to stimulatory Gα protein to drive cAMP production ^190^. Biliary exosomes in the bile interact with cilia to suppress ERK-mediated proliferation, whilst cholangiocytes without cilia do not respond to exosomes and proliferate ^191^. In the case of loss of *Pkd1* or *Pkd2* in the mouse, development of ADPKD depends on intact cilia; inappropriate signalling mediated by cilia upon disruption of PKD1/2 leading to cysts can be partially rescued by ablating cilia via *Ift20* and/or *Kif3a* knockout ^192^. In postnatal mouse cholangiocytes, cilia loss leads to cystogenesis through an upregulation of a paracrine TGFB/R-SMAD proinflammatory axis, which remodels the cystic niche and biophysical properties of cystic ducts ^193^. Here, cilia seem to regulate the size, shape and mechanical properties of the committed bile ducts. From a translational perspective, remarkable recent work in mice has shown that similar aberrant changes in cell morphology, lumen size and extracellular matrix deposition of end-stage cystic kidneys can be reversed by restoring ADPKD gene function ^194^.

## Conclusions and outlook

**Figure 4:**
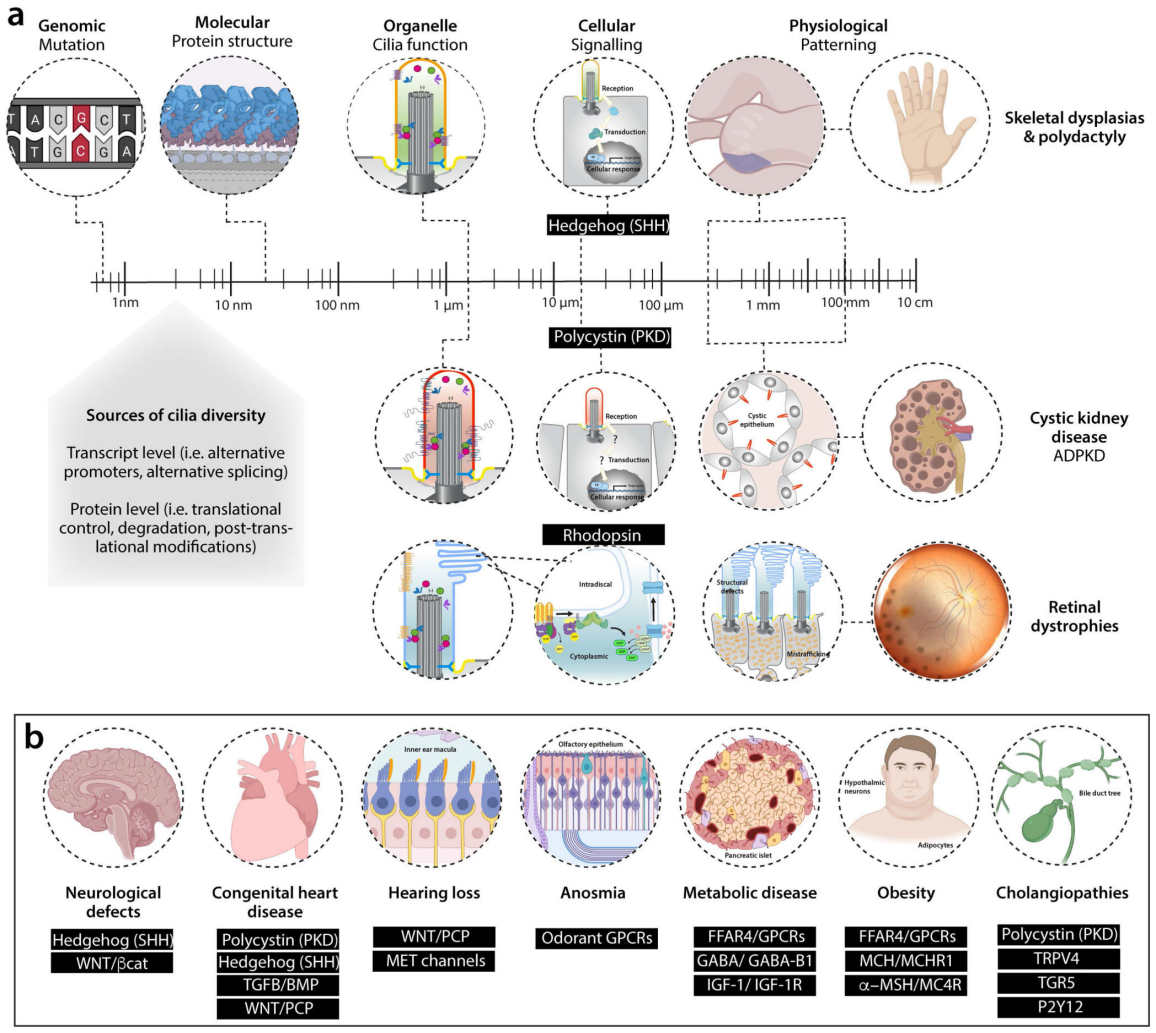
Challenge of ciliopathies — biology across scales. **a**, Schematic of how variant identification in a patient with ciliopathy is just the start of the challenge. Genetic changes are the same across all cell types. Understanding how these variants disturb different types of cilia structurally and functionally in terms of signalling readouts is our knowledge gap across cell types and developmental times, one that we need to address to understand patient phenotypes. Possible sources of tissue-specific phenotypes are highlighted. We focus on the best characterized cilia-dependent signalling defects resulting from ciliopathy mutations in different tissue types, including skeleton, kidney epithelia and photoreceptors resulting in ciliopathic disease. For the kidney, we have focused on autosomal dominant polycystic kidney disease through PKD1/2 signalling but other renal diseases include autosomal recessive polycystic kidney disease and nephronophthisis, the latter involving additional signaling defects (see Table 1). **b**, Examples of the signalling pathways disrupted in different tissue types in the ciliopathies. Abbreviations: ADPKD, autosomal dominant polycystic kidney disease; FFAR4, Free Fatty Acid Receptor 4; GABA, gamma-aminobutyric acid; GPCRs, G protein coupled receptors; IGF, insulin-like growth factor; MCH, melanin-concentrating hormone; MET, mechano-electrical transduction; MSH, melanocyte-stimulating hormone; P2Y12, purinergic receptor P2Y; PKD, Polycystin; SHH, Sonic Hedgehog; TGFB/BMP, Transforming Growth Factor Beta/ Bone Morphogenetic Protein; TGR5, Takeda G protein-coupled receptor 5; TRPV4, transient receptor potential vanilloid-type 4; WNT/ βcat, canonical Wingless/Integrated Beta catenin pathway; WNT/PCP, non-canonical Wingless/Integrated Planar Cell Polarity pathway.

Once thought of as evolutionary remnants ^195^, primary cilia are known to play essential roles as coordinators of multiple signalling pathways that control key cellular processes during development and homeostasis of tissues and organs. Pioneered by early studies on IFT that provided the first links between primary cilia, vertebrate signalling and development and disease ^196–198^, a large body of literature has since substantiated the importance of primary cilia in human health and disease, with more than 30 distinct ciliopathies and 247 ciliopathy disease genes identified to date ^2,6,7^. Despite impressive recent progress in identifying genetic variation and dissecting the molecular aetiology of ciliopathies, understanding the correlation between genotype and clinical phenotypes of patients with ciliopathies remains an important challenge in the field — one of biology across scales (Figure 4). Moreover, how defective primary cilia can give rise to isolated, single organ diseases or syndromic ciliopathies affecting multiple organs remains unclear; it suggests not all cilia are ‘equal’, resulting in differences in sensitivity to dysfunction through mutation

Importantly, we need to tackle the complexity of cilia-mediated signalling in vivo at the intracellular level (for example, cross-talk between signalling pathways), at the level of specific cell types, and across tissues or centres to integrate complex physiological circuits (Figure 4a). To this end, we need better tools to track structural and functional diversity of primary cilia in biologically relevant cell types across developmental times (Figure 4b). Recent technological developments, including various omics approaches, have provided detailed insight into the ciliary signalling repertoire and its changes in response to extracellular signals or in specific mutant backgrounds ^89,90,94,133^. However, knowledge of differences in ciliary structure, composition and signalling function *in vivo* across cell types, tissues and environmental or developmental contexts remains scarce.

In this era of functional genomics, we need improved means to capture cell- and stage-type specific differences in expression of isoforms. At the mRNA level, alternative splicing or promoters could be captured via single-cell and spatial transcriptomics ^199^. The importance of post-transcriptional regulation is evidenced by human mutations in pre-mRNA processing factor genes (*PRPF3/4/6/8/31* (OMIM: 607301, 607795, 613979, 607300, 606419), *SNRNP200* (OMIM: 601664) and *RP9* (OMIM: 607331)), which have been linked to 15–20% of autosomal dominant ^200^ retinitis pigmentosa. However, as these factors are ubiquitously expressed, most tissues seem to tolerate defective splicing, whilst the neuroretina is highly sensitive. We also need to tackle differences at the proteome level, to monitor differential translational and/or degradation programmes in different tissues, some of which have been shown to be regulated locally near cilia or centrosomes ^201,202^. Exciting advances in spatial proteomics, such as LOPIT-DC (Localisation of Organelle Proteins by Isotope Tagging after Differential ultracentrifugation) ^203^, which enables identification of isoform-specific localizations and assignment of proteins to suborganellar structures, may help to resolve differences in ciliary apparatus and centrosomes between cell types. Other new techniques for preserving tissue spatial context include DVP (Deep Visual Proteomics) ^204^, which combines a novel artificial intelligence-driven phenotype image analysis tool with single-cell laser microdissection and ultra-high-sensitivity mass spectrometry. Here, protein abundance is linked to complex cellular or subcellular phenotypes whilst preserving spatial context, say in adjacent cystic versus non-cystic epithelium in polycystic kidney disease, as well as associated pathological changes in the cystic niche, remodelled fibroblasts and inflammatory infiltrates.

A technical challenge remains in applying omics approaches more broadly to physiologically relevant tissues *ex vivo* or ideally *in vivo*. To maximally capitalize on the exquisite spatial and temporal resolution of proteomes, with APEX2 proximity labelling, for example, we need to overcome current limitations in cells with abundant endogenous peroxidases as well as sensitivity to H_2_O_2_ using synthetic biology and biorthogonal chemistry to evolve both better enzymes and specific substrates ^205,206^. In the future, extending current implementations of technologies such as proximity-labelling proteomics may allow for cell-, tissue- and developmental-stage-specific monitoring of cilia composition, providing insight into mechanisms underlying specific context-dependent roles for cilia and ciliary proteins *in vivo*. These studies may reveal differences in sensitivity to cilia dysfunction between tissues or stages, which could help to better understand pathomechanisms underlying ciliopathies.

In parallel, recent advances in imaging technologies ^53^ and optogenetics ^207–209^ can be harnessed to dissect ciliary structure and signalling function not only *in vitro* but also *in vivo*, such as in a recently reported, elegant study identifying a serotonergic axon–cilium synapse in the mouse brain ^209^. With respect to signalling, we need better imaging modalities that capture the highly dynamic (less than a second) nanoscale (tens of nanometers) events that underlie compartmentalized signalling, such as GPCR signalling within a cilium ^210^. Technical breakthroughs towards improved temporal resolution in single-molecule localization microscopy (SMLM) through development of faster, improved signal-to-noise, high-speed cameras than current EMCCD and sCMOS technology ^211^ as well as improved fluorophore photon yields and membrane permeability will be important for this field. New means to build better reporters using genome editing of endogenous loci and non-disruptive tagging approaches are also key. These could include expansion of the genetic code, permitting incorporation of synthetic amino acids into ciliary proteins, allowing functionalization through click chemistry ^212^. Together with new workflows, tools and biosensors, the field could begin to understand how signalling initiated from nanoscale complexes within cilia is propagated across the organelle, cell and tissue levels during development and how this flow is disrupted in disease.

Finally, adding another layer of complexity to our current view of the ciliary signalling repertoire, recent evidence based primarily on cultured cells of non-vertebrate model organisms has suggested that cilia may not only function as receivers of extracellular signals, but also emit signal to other cells in the form of extracellular vesicles ^126^. Determining the extent and physiological importance of this phenomenon *in vivo* will be an important goal for the future.

## Figures and Tables

**Table 1:** Established ciliopathies and associated disease genes

Name^a^	Commonly observed clinical features^b^	Known disease gene(s)^c^
Acrocallosal syndrome (ACLS)	Agenesis of corpus callosum, distal anomalies of limbs, minor craniofacial anomalies and intellectual disability.	***KIF7, GLI3***
Alström syndrome (ALMS)	Vision and hearing abnormalities, childhood obesity, cardiomyopathy. Later in life diabetes mellitus, liver and kidney dysfunction may develop.	***ALMS1***
Bardet-Biedl syndrome (BBS)	Cone-rod dystrophy, polydactyly, truncal obesity, hypogonadism, kidney abnormalities, learning difficulties, congenital heart defects, cardiomyopathy.	***ARL6, BBPIP1, BBS1, BBS2, BBS4, BBS5, BBS7, BBS9, BBS10, BBS12, C8ORF37, CEP290, IFT27, IFT74, IFT172, LZTFL1, MKKS, MKS1, SDCCAG8, TRIM32, TTC8, WDPCP***
Carpenter syndrome (CRPT)	Craniosynostosis, skeletal and dental abnormalities, vision and hearing loss, congenital heart defects, genital abnormalities, obesity, intellectual disability.	***MEGF8, RAB23***
Cerebellar vermis defect, oligophrenia, ataxia, coloboma, hepatic fibrosis (COACH) syndrome	Intellectual disability, liver fibrosis, ataxia, ocular anomalies (coloboma, nystagmus). Considered a rare form of Joubert syndrome.	***CC2D2A, RPGRIP1L, TMEM67***
Cranioectodermal dysplasia (CED; also known as Sensenbrenner syndrome)	Skeletal and ectodermal defects, nephronophthisis, liver fibrosis, ocular anomalies (mainly retinitis pigmentosa), congenital heart defects	***IFT43, IFT122, WDR19 (IFT144), WDR35 (IFT121)***
Curry–Jones syndrome (CRJS)	Syndromic craniosynostosis, agenesis of the corpus callosum, preaxial polysyndactyly and syndactyly of hands and/or feet, skin and intestinal abnormalities, ocular anomalies (colobomas, microphthalmia), occipital meningoceles, intellectual disability, tumours (smooth muscle hamartomas, desmoplastic medulloblastoma).	***SMO***
Ellis–Van Creveld (EVC) syndrome	Short stature, short arms and legs, narrow chest with short ribs, polydactyly, missing and/or malformed nails, dental abnormalities, congenital heart defects.	***EVC, EVC2***
Endocrine-cerebroosteodysplasia (ECO)	Various anomalies of the endocrine, cerebral, and skeletal systems, neonatal mortality.	***CILK1***
Greig cephalopolysyndactyly syndrome (GCPS)	Polydactyly, syndactyly, ocular hypertelorism, macrocephaly, intellectual disability.	***GLI3***
Holoprosencephaly (HPE)	Abnormal brain development, cyclopia, proboscis, intellectual disability, pituitary gland anomalies.	*CDON, **FGF8, FOXH1, GLI2**, NODAL, **PTCH1, SHH,** SIX3, TGIF1, ZIC2*
Hydrolethalus syndrome (HLS)	Severe foetal malformations including craniofacial dysmorphic features and abnormalities of central nervous system, heart, respiratory tract and limbs.	***HYLS1, KIF7***
Jeune asphyxiating thoracic dystrophy (JATD)/ (also known as shortrib thoracic dysplasia (SRTD))	Defective bone development, including small chest and short ribs causing impaired growth and expansion of the lungs and breathing difficulties; shortened bones in the arms and legs, polydactyly, unusually shaped pelvic bones.	***CEP120, DYNC2H1, DYNC2I1 (WDR60), DYNC2I2 (WDR34), DYNC2LI1, DYNLT2B, IFT43, IFT52, IFT80, IFT81, IFT140, IFT172, INTU, KIAA0586 (TALPID3), KIAA0753 (MNR), NEK1, TCTEX1D2, TTC21B (IFT139), WDR19 (IFT144), WDR35 (IFT121)***
Joubert syndrome (JBTS)	Defective brain development, including absence or underdevelopment of the cerebellar vermis and a malformed brain stem, which cause the characteristic molar tooth sign on MRI. Congenital heart defects. Other symptoms include hypotonia, abnormal breathing patterns and eye movements, ataxia, distinctive facial features, and intellectual disability.	***AHI1, ARL13B, ARL3, ARMC9, B9D1, B9D2, CC2D2A, CEP41, CEP104, CEP120, CEP290, CPLANE1, CSPP1, FAM149B1*** ^213,214^, ***IFT74, INPP5E, KATNIP*** ^215^,***KIAA0586 (TALPID3), KIAA0753 (MNR), KIF7, MKS1, NPHP1, OFD1, PDE6D, PIBF1, RPGRIP1L, SUFU, TCTN1, TCTN2, TCTN3, TMEM67, TMEM107, TMEM138, TMEM216, TMEM218*** ^216^, ***TMEM231, TMEM237, TOGARAM1, TTC21B (IFT139),*** *ZNF423*
Kallmann syndrome (KS: central hypogonadism)	Hypogonadotropic hypogonadism leading to impaired sexual development; impaired sense of smell.	>50 genes; see Ref. ^217^
Leber congenital amaurosis (LCA)	Retinal defects, causing severe visual impairment beginning in infancy. Other symptoms include photophobia, nystagmus, keratoconus and extreme farsightedness.	***AIPL1, ALMS1, CEP290, CNGA3*** ^218^, *CRB1, CRX, DTHD1, GDF6, GUCY2D, IDH3A, IMPDH1, **IQCB1 (NPHP5), KCNJ13, LCA5,** LRAT, **MYO7A**, NMNAT1, PHPH2, RD3, RDH12, RPE65, **RPGRIP1, SPATA7, TUBB4B, TULP1,** USP45*
McKusick–Kaufman syndrome (MKKS)	Genitourinary malformations, postaxial polydactyly, congenital heart defects, choanal atresia, pituitary dysplasia, esophageal atresia and distal tracheoesophageal fistula, Hirschsprung disease, vertebral anomalies, and hydrops fetalis. Syndrome is allelic with Bardet-Biedl.	***MKKS***
Meckel syndrome (MKS)	Multiple kidney cysts, occipital encephalocele, polydactyly, congenital heart defects. Affected children may also display anomalies of head, face, liver, lungs, genitals, and urinary tract.	***B9D1, B9D2, CC2D2A, CEP290, KIF14, MKS1, NPHP3, RPGRIP1L, TCTN2, TMEM67, TMEM107, TMEM216, TMEM231**, TXNDC15*
Mental retardation, truncal obesity, retinal dystrophy, and micropenis syndrome (MORMS)	Intellectual disability, truncal obesity, retinal dystrophy, and micropenis in males. Cataracts may occur later in life.	***INPP5E***
Morbid obesity and spermatogenic failure (MOSPGF)	Morbid obesity, hypertension, type 2 diabetes mellitus and dyslipidemia leading to early coronary disease, myocardial infarction and congestive heart failure; intellectual disability, decreased sperm counts or azoospermia.	***CEP19***
Nephronophthisis (NPHP)	Renal dysfunction, chronic tubulointerstitial nephritis, renal cyst formation and progression to end stage renal disease. Congenital heart defects, cardiomyopathy.	***ANKS6, CEP83, CEP164, CEP290, DCDC2, GLIS2, IFT172, INVS, IQCB1, MAPKBP1*** ^219^, ***NEK8, NPHP1, NPHP3, NPHP4, RPGRIP1L, SDCCAG8, TMEM67, TTC21B (IFT139), WDR19 (IFT144),*** *ZNF423*
Oculocerebrorenal syndrome of Lowe (OCRL)	Defects in eyes, central nervous system and kidneys; hypotonia and feeding difficulties, developmental delay, intellectual disability, behavioural problems, seizures and short stature. Occurs almost exclusively in males.	***OCRL***
Oral-facial-digital syndrome (OFDS)	Defective development of brain, heart, face, limbs and kidneys; polycystic kidneys.	***C2CD3, C5orf42, CPLANE1, DDX59, IFT57, INTU, KIAA0753 (MNR), NEK1, OFD1, SCLT1, SCNM1, TBC1D32, TCTN3, TMEM107, TMEM138, TMEM216, TMEM231, WDPCP***
Pallister-Hall syndrome (PHS)	Polydactyly, syndactyly, hypothalamic hamartoma, and bifid epiglottis. Other symptoms include imperforate anus, abnormalities in the kidneys, cardiac defects, small genitalia, lack of fingers, nail problems, cleft palate, bifid uvula, and development delay and behavioural problems.	***GLI3***
Pituitary stalk interruption syndrome (PSIS)	Congenital abnormality of the pituitary leading to pituitary deficiency.	*CDON, **IFT56 (TTC26)*** ^220^, ***GPR161**, HESX1, LHX4, PROKR2, ROBO1, **WDR11***
Polycystic kidney/liver disease (PKD)	Enlarged cystic and dysfunctional kidneys and/or livers.	*ALG5, ALG8, DNAJB11, **DZIP1L**, GANAB, JAG1, LRP5, **PKD1, PKD2**, *PKHD1*, PRKCSH, SEC63,*
Retinitis pigmentosa (RP)	Retinal defects leading to progressive vision loss.	>90 genes; see RetNet, the Retinal Information Network
RHYNS syndrome	Syndromic retinal disorder characterized by the association of retinitis pigmentosa, hypopituitarism, nephronophthisis, and skeletal dysplasia.	***TMEM67*** ^221^
Senior–Løken syndrome (SLSN)	Nephronophthisis (NPHP) associated with retinal dystrophy.	***CEP164, CEP290, INV, IQCB1, NPHP1, NPHP3, NPHP4, SDCCAG8, TRAF3IP1, WDR19 (IFT144)***
Stromme syndrome (STROMS)	Usually characterized by microcephaly, ocular anomalies, and apple-peel intestinal atresia. Other symptoms include facial dysmorphism, motor delay and intellectual disability, as well as heart, brain, kidney, and craniofacial abnormalities.	***CENPF***
Syndactyly-telecanthus-anogenital and renal malformations (STAR) syndrome	Syndactyly, telecanthus, anogenital and renal malformations.	***CCNQ*** ^222^
Usher (USH) syndrome	Sensorineural hearing loss or deafness and progressive vision loss due to retinitis pigmentosa.	***ADGRV1**, ARSG, CDH23, **CEP78**^d^, **CEP250**, CIB2, **CLRN1**, DFNB31, ESPN, HARS1, **MYO7A, PCDH15, PDZD7**, USH1C, **USH1G, USH2A, WHRN***
Von Hippel-Lindau (VHL) disease	Abnormal growth of both benign and cancerous tumours and cysts in many parts of the body, including central nervous system, kidney, pancreas, adrenal glands and endolymphatic sac. Anxiety disorders.	***VHL***
Weyer acrodental dysostosis (WAD)	Milder form of Ellis-Van Creveld syndrome without congenital heart defects.	***EVC, EVC2***

a The list of ciliopathy disease genes was modified from Ref.^7^.

b Adapted from Genetic and Rare Diseases Information Center (GARD) and Orphanet.

c Unless otherwise indicated, the genes listed were obtained by searching Ref.^7^ and the OMIM database using the disease name as search entry. Genes indicated in bold encode proteins localizing to the cilium–centrosome axis according to the SYSCILIA gold standard version 2 ^223^, or alternative studies as indicated for some genes, where supporting references are indicated.

d Studies have suggested that patients with *CEP78* mutations can present with atypical Usher syndrome or retinitis pigmentosa ^224^.

## Box 1 Intraflagellar transport.

Intraflagellar transport (IFT) refers to the conserved microtubule-based motility system that moves bidirectionally along ciliary axonemes to transport cargoes into and out of the organelle. Originally identified in the green alga *Chlamydomonas reinhardtii* ^225^, IFT has subsequently been identified in all ciliated eukaryotic organisms, including protists, worms, and vertebrates ^8,226,227^.

Canonical IFT consists of anterograde (heterotrimeric kinesin-2) and retrograde (cytoplasmic dynein 2) microtubule motors that move trains of IFT-A and IFT-B complexes with associated cargoes from the ciliary base to the tip (kinesin-2) and back (dynein 2). Transport of axonemal building blocks, for example, tubulin, to the ciliary tip occurs through direct binding to specific IFT proteins ^228–230^, whereas IFT-mediated retrograde transport of ciliary integral or peripheral membrane proteins relies on the BBSome as cargo adaptor ^25,231^. As for anterograde IFT of transmembrane cargo, this is thought to occur by a mechanism involving both IFT-A and associated TUBBY-like proteins, which have also been implicated in ciliary import of lipidated cargoes ^25,28–30,97^. In some cell types and organisms, additional kinesins such as homodimeric kinesin-2 (OSM-3 in *Caenorhabditis elegans*, KIF17 in humans) and kinesin-3 (KLP-6 in *C*. *elegans*, KIF13B in humans) motors function in coordination with canonical IFT to modulate ciliary structure and function ^232,233^.

Numerous studies performed in different cell types and organisms have shown that IFT is required for assembly and function of almost all eukaryotic cilia and flagella ^227,232,234^. Fundamental studies on IFT were instrumental in providing the first link between primary cilia, developmental signalling and human disease ^196–198^, and mutations in IFT genes are now known to be causative of multiple ciliopathies ^2,8^.

Recent advances in imaging and structural biology, combined with molecular, genetic and biochemical approaches, have provided detailed insight into the 3D structure and molecular workings of the core IFT system ^8,30,33,97,232,234–236^, as well as the IFT-A-associated Tubby-like proteins and retrograde membrane cargo adaptor complex, the BBSome ^29,30,237–239^ (see the table for a list of IFT components).

**Table:**

Complex	Protein name	Alternative name(s)	Gene	OMIM number
Heterotrimeric kinesin-2 (canonical anterograde motor)	KIF3A	NA	*KIF3A*	604683
	KIF3B	KIAA0359	*KIF3B*	603754
	KIF3AP	KAP3	*KIF3AP*	601836
Homodimeric kinesin 2 (accessory anterograde motor)	KIF17	KIAA1405	*KIF17*	605037
Cytoplasmic dynein 2 (retrograde motor)	DYNC2H1	DNCH2, DHC2, DHC1B	*DYNC2H1*	603297
	DYNC2I1	WDR60	*DYNC2I1*	615462
	DYNC2I2	WDR34	*DYNC2I2*	613363
	DYNC2LI1	D2LIC, LIC3	*DYNC2LI1*	617083
	DYNLL1	DNCL1, DLC1, PIN, LC8	*DYNLL1*	601562
	DYNLL2	DLC2	*DYNLL2*	608942
	TCTEX1D2	NA	*DYNLT2B*	617353
	DYNLT1	TCTEL1	*DYNLT1*	601554
	DYNLT3	TCTE1L	*DYNLT3*	300302
	DYNLRB1	DNCL2A, DNLC2A	*DYNLRB1*	607167
	DYNLRB2	DNLC2B	*DYNLRB2*	607168
IFT-B1	IFT88	TG737, Polaris, TTC10, DAF19	*IFT88*	600595
	IFT81	CDV1	*IFT81*	605489
	IFT74	CCDC2, CMG1, BBS22, JBTS40, SPGF58	*IFT74*	608040
	IFT70	TTC30B	*TTC30B*	NA
	IFT56	TTC26, BRENS	*TTC26*	617453
	IFT52	SRTD16	*IFT52*	617094
	IFT46	C11orf2	*IFT46*	NA
	IFT27	RABL4, BBS19	*IFT27*	615870
	IFT25	HSPB11	*HSPB11*	NA
	IFT22	RABL5	NA	NA
IFT-B2	IFT172	SLB, KIAA1179, BBS20, RP71, SRTD10	*IFT172*	607386
	IFT80	KIAA1374, WDR56, SRTD2	*IFT80*	611177
	IFT57	ESRRBL1, HIPPI, OFD18	*IFT57*	606621
	IFT54	TRAF3IP1, MIPT3, SLSN9	*TRAF3IP1*	616629
	IFT38	CLUAP1, FAP22, DYF3, QILIN	*CLUAP1*	616787
	IFT20	None	*IFT20*	614394
IFT-A1	IFT144	WDR19, SPGF72, CED4, NPHP13, SLSN8, SRTD5	*WDR19*	608151
	IFT140	KIAA0590, RP80, SRTD9	*IFT140*	266920
	IFT122	WDR10, CED1	*IFT122*	606045
IFT-A2	IFT139	TTC21B, THM1, NPHP12, SRTD4	*TTC21B*	612014
	IFT121	WDR35, KIAA1336, CED2, SRTD7	*WDR35*	613602
	IFT43	C14ORF179, CED3, RP81, SRTD18	*IFT43*	614068
BBSome (retrograde IFT-B membrane cargo adaptor)	BBS1	NA	*BBS1*	209901
	BBS2	RP74	*BBS2*	606151
	BBS4	NA	*BBS4*	600374
	BBS5	NA	*BBS5*	603650
	BBS7	FLJ10715	*BBS7*	607590
	BBS8	TTC8, RP51	*TTC8*	615985
	BBS9	PTHB1	*BBS9*	615986
	BBS18	BBIP1	*BBIP1*	615995

Names refer to human proteins/genes. NA, not available.

### Related links

GARD https://rarediseases.info.nih.gov/

OMIM https://www.omim.org/

610142, 610937, 605446, 609863, 613846, 604730, 601197, 601313, 173910, 601309,

601500, 608922, 613037, 608002, 604695, 602676, 604011, 605413, 615519

604683, 603754, 601836, 605037, 603297, 615462, 613363, 617083, 601562, 608942,

617353, 601554, 300302, 607167, 607168, 600595, 605489, 608040, 617453, 617094,

615870, 607386, 611177, 606621, 616629, 616787, 614394, 608151, 266920, 606045,

612014, 613602, 614068, 209901, 606151, 600374, 603650, 607590, 615985, 615986,

615995

Orphanet https://www.orpha.net/consor/cgi-bin/index.php

RetNet https://web.sph.uth.edu/RetNet/sum-dis.htm#A-genes

## Glossary terms

Axoneme: Core structure of cilia, which usually comprises 9 outer microtubule doublets and 0 or 2 central microtubule singlets in primary (9 + 0) and motile cilia (9 + 2), respectively.
Basal body: Specialized centriole that provides the foundation of the axoneme and anchors the cilium to the cell body via distal and subdistal appendages or fibres.
BBSome: A complex composed of eight Bardet–Biedl syndrome proteins, which is thought to function as a cargo adaptor for the retrograde IFT machinery during ciliary membrane protein export.
Centriole: Barrel-shaped structure composed of 9 microtubule triplets that gives rise to the basal body and constitutes the core structure of the centrosome.
Centrosome: Main microtubule organizing centre in animals. Composed of two centrioles (mother and daughter) surrounded by pericentriolar material.
Ciliopathy: A genetic disorder characterized by ciliary dysfunction, and whose affected gene product localizes to (first-order ciliopathy) or indirectly affects (second-order ciliopathy) the cilium–centrosome axis.
Ciliary necklace: Outer aspect of the ciliary membrane located at the transition zone region where Y-links connect the inside of the membrane to the axoneme.
Ciliary pocket: An invagination of the periciliary membrane, which is a hotspot for endocytosis and exocytosis of vesicles derived from or destined to the ciliary membrane.
Ectosomes: Extracellular vesicles that bud directly from the membrane, including the ciliary membrane.
Intraflagellar transport: (IFT) Conserved intraciliary transport system in which kinesin-2 and cytoplasmic dynein 2 motors move trains of cargo-associated IFT-A/B complexes into and out of cilia. Required for ciliary assembly, maintenance and function.
Periciliary membrane: Membrane region at the base of the cilium that connects the ciliary membrane with the plasma membrane. Sometimes invaginated to form a ciliary pocket.
Phosphoinositides: (PIPs) Lipid signalling molecules that coordinate multiple membrane-associated molecular events.
Primary cilium: Non-motile sensory organelle present on the cell surface. It typically has a 9 + 0 axoneme surrounded by a membrane enriched for specific receptors and ion channels involved in signalling.
Transition zone (TZ): Ciliary sub compartment located between the basal body and cilium itself, which functions as a barrier for selective transport of molecules into and out of the cilium.
